# MINERVA—a platform for visualization and curation of molecular interaction networks

**DOI:** 10.1038/npjsba.2016.20

**Published:** 2016-09-22

**Authors:** Piotr Gawron, Marek Ostaszewski, Venkata Satagopam, Stephan Gebel, Alexander Mazein, Michal Kuzma, Simone Zorzan, Fintan McGee, Benoît Otjacques, Rudi Balling, Reinhard Schneider

**Affiliations:** 1 Luxembourg Centre for Systems Biomedicine, Université du Luxembourg, Esch-sur-Alzette, Luxembourg; 2 European Institute for Systems Biology and Medicine, Université de Lyon, eTRIKS Consortium, Lyon, France; 3 Institute of Computing Science, Poznan University of Technology, Poznan, Poland; 4 Luxembourg Institute of Science and Technology, Belvaux, Luxembourg

## Abstract

Our growing knowledge about various molecular mechanisms is becoming increasingly more structured and accessible. Different repositories of molecular interactions and available literature enable construction of focused and high-quality molecular interaction networks. Novel tools for curation and exploration of such networks are needed, in order to foster the development of a systems biology environment. In particular, solutions for visualization, annotation and data cross-linking will facilitate usage of network-encoded knowledge in biomedical research. To this end we developed the MINERVA (Molecular Interaction NEtwoRks VisuAlization) platform, a standalone webservice supporting curation, annotation and visualization of molecular interaction networks in Systems Biology Graphical Notation (SBGN)-compliant format. MINERVA provides automated content annotation and verification for improved quality control. The end users can explore and interact with hosted networks, and provide direct feedback to content curators. MINERVA enables mapping drug targets or overlaying experimental data on the visualized networks. Extensive export functions enable downloading areas of the visualized networks as SBGN-compliant models for efficient reuse of hosted networks. The software is available under Affero GPL 3.0 as a Virtual Machine snapshot, Debian package and Docker instance at http://r3lab.uni.lu/web/minerva-website/. We believe that MINERVA is an important contribution to systems biology community, as its architecture enables set-up of locally or globally accessible SBGN-oriented repositories of molecular interaction networks. Its functionalities allow overlay of multiple information layers, facilitating exploration of content and interpretation of data. Moreover, annotation and verification workflows of MINERVA improve the efficiency of curation of networks, allowing life-science researchers to better engage in development and use of biomedical knowledge repositories.

## Introduction

Biomedical knowledge is rapidly growing in size and complexity. Many repositories have been established to complement the literature body of knowledge by encoding and providing access to the curated information on molecular interactions. Both the literature and repositories support the effort of knowledge curation—the process of gathering relevant information pieces and assembling them into a contextualized molecular interaction network. Such curated networks may greatly help in the analysis and interpretation of highly dimensional datasets produced by modern biomedical assays.^1^

Experts creating a mechanistic description of molecular networks face the challenges of format standardization, and need support in quality control of generated networks, their exploration, use and sharing. The first challenge is well addressed by existing and continuously updated process description languages, such as Systems Biology Graphical Notation.^2^ In order to tackle the remaining issues, we have developed the MINERVA (Molecular Interaction NEtwoRks VisuAlization) platform.

The management of biomedical knowledge in the form of molecular networks requires a number of components. First, easy access to the repository is needed, as network content is often assembled by entire groups of researchers over time, like in WikiPathways or Parkinson’s disease map.^3,4^ Second, comprehensive visualization and exploration of complex content are required in order to grasp high-level associations and access single mechanism-level details, even for large networks. Third, capabilities for detailed content curation and extraction, aided by automatic annotation and verification, are mandatory in case of large, longitudinally maintained repositories. Currently available solutions address these challenges to a significant extent, but have certain limitations regarding their integration as a user-centric service.

The MINERVA platform integrates the functionalities of pathway databases and user-centric content hosting into a standalone web-based platform for knowledge repositories of molecular interaction networks. It features extensive interactivity with the content, and straightforward and automated annotation of hosted networks. MINERVA enables highlighting targets of drugs, and the upload and visualization of user-provided experimental datasets, including multi-omic entries and custom color coding.

Importantly, our platform supports content management and re-use. It handles both CellDesigner and SBGN formats and gives users the ability to directly download chosen areas of the visualized diagram. A commenting system enables users to directly annotate the explored content, providing direct feedback to the curators of MINERVA repositories. MINERVA allows web management of hosted maps, registered users, recorded comments and interfaces to external bioinformatic databases.

In this article, we describe the MINERVA platform and its functionalities. We briefly review repositories of molecular interactions and their supporting technologies. Next, we provide details on the architecture and important functions of our solution. Then, we focus on use-case description for knowledge repositories supported by the MINERVA platform. Finally, we describe further steps foreseen in the development of the platform.

## Related work

Repositories of molecular interaction networks vary in size and scope. Large-scale databases of molecular interactions, such as PSICQUIC^5^ or IntAct,^6^ offer a broad insight into the function of a given molecule. Construction of networks from such sources provides an excellent basis for network analysis and unbiased hypothesis generation. In order to construct detailed hypotheses on the mechanisms of biological systems, further refinement of such networks is required. One of the possible approaches is to assemble contextualized groups of interactions into focused, diagrammatically represented graphs, called pathways.

Pathway repositories, such as Reactome,^7^ PANTHER,^8^ KEGG^9^ and WikiPathways,^3^ enable easy access to carefully curated molecular interaction networks and provide search engines allowing to explore it. The curation process, ensuring high-quality content, can be driven by dedicated curators, like in the case of Reactome or KEGG, or involve community experts, like WikiPathways. Remarkably, the content offered by pathway databases becomes more and more interoperable, allowing its efficient re-use, with SBGN becoming a common denominator in many cases. For instance, Reactome and PANTHER follow SBGN in its visual layout, and provide export to this format. Similarly, WikiPathways-supporting pathway editor PathVisio^10^ offers SBGN export among many others. Finally, a workflow for translation of KEGG pathways into SBGN format was recently proposed.^11^ Such pathway data interoperability greatly facilitates development of molecular interaction networks aimed for specific research project, or a focused knowledge domain.

Currently, individual researchers or research consortia gain the possibility to curate literature- and pathway-derived information snippets into specialized molecular interaction networks. Such dedicated knowledge repositories, constructed with various editors, including CellDesigner,^12^ PathVisio or SBGN-ED (http://sbgn-ed.org), need to be supported with visualization and data-mapping frameworks to facilitate knowledge exploration and sharing. A number of solutions were proposed to address this challenge, either by handling diagram visualization using dedicated technology, like Payao^13^ and BioUML,^14^ or by relying on Google Maps API, like CellPublisher.^15^ A notable example in this domain is the recently proposed platform NaviCell.^16^

NaviCell is a platform oriented on hosting CellDesigner diagrams using Google Maps API. It provides functionalities similar to pathway databases—content annotation, interactive exploration and network-based data visualization^17^—but for user-drawn CellDesigner diagrams.^18^ With the support of GoogleMaps API, the platform enables interaction with elements of displayed diagrams. It is worth noting that the 'map staining' approach to visualization of experimental data is a compelling idea for comprehensive representation of high-dimensional datasets.

In the light of the increasing interoperability of molecular interaction data, and growing knowledge on mechanisms of biological systems, it is important to continue the development of technologies supporting contextualized systems biology repositories. Of particular importance are areas of curation support, and knowledge sharing and re-use.

## Results

### Automatic annotation using online bioinformatics resources

The platform features configurable automated annotation tools. These annotators use either the name of an element or its MIRIAM identifier, fetch automatically information from a corresponding database, and update annotations of elements in the uploaded network. Currently available annotators use the following:

- Element name: BioCompendium (http://biocompendium.embl.de) and HGNC.^19^
-MIRIAM identifier: ChEBI,^20^ Ensembl,^21^ Entrez,^22^ Uniprot^23^ and Gene Ontology.^24^

Annotated resources are hyperlinked to the corresponding online resources and are immediately accessible for interactive exploration.

A set of rules for automatic verification of the annotation of uploaded content can be configured using the administrator panel. Warning messages are displayed when annotation type requirements are not fulfilled. For instance, when a ‘PubMed’ field is required for the annotation of interactions, warnings will be generated for each interaction, which has no PubMed annotation. Such a rule-based control supports quality and completeness checks of the uploaded models, which is especially important for large diagrams.

### Visualization of structured content via GoogleMaps API

Upon upload, molecular interaction networks in the SBGN-compliant format are translated to the data model and uploaded to the database. The visualization is generated using the Google Maps API. Importantly, the visualization provided by the MINERVA platform is interactive for both elements and interactions. Annotation on the elements and interactions are visible in the left panel of the interface. The contents are searchable, and multiple-element queries are allowed. Different queried elements, if present in the map, will be indicated with markers of different color, allowing the identification of areas where two elements may belong to the same process or pathway.

For visualized maps, MINERVA generates automatically a semantic zoom. In the uploaded diagram, compartments, complexes and text areas are identified. An algorithm processes these areas, and on the basis of their size and coverage assigns them to appropriate zoom levels in such a way that larger areas cover smaller ones. In this automatically created view, a zoomed-out map displays only the most generic areas, which reveal more detailed views with incrementing zoom-in.

MINERVA enables the construction and visualization of complex molecular interaction maps, with a structure of nested submaps. The platform accepts as an input a bundle of multiple files, which can be uploaded together with a configuration file, describing relationships of the remaining maps. The configuration file itself is a diagram drawn in CellDesigner, indicating elements that act as cross-map hyperlinks, and interactions denote relationships between the maps. This allows construction of flexible relationships between submaps, and supports compartmentalization of large maps.

### Functionalities for content exploration

#### Online queries for drug targets via DrugBank and CHEMBL

**Figure 1:**
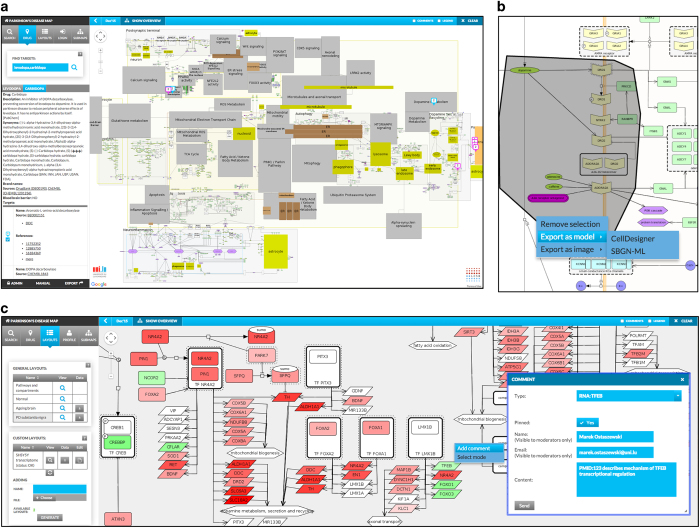
MINERVA interface and functionalities. (**a**) Main interface, displaying drug target search results for terms ‘levodopa’ and ‘carbidopa’. Information fetched from DrugBank and ChEMBL are displayed in the left panel, while targets of queried drugs are shown in the display area as markers. (**b**) Export of the selected content. A portion of the diagram is selected and exported as a model (CellDesigner or SBGN formats). (**c**) Display of experimental data and content commenting. Publicly available datasets (left panel, ‘General overlays’) or user-provided datasets (left panel, ‘User-provided overlays’) can be visualized on top of the displayed content. Users can pin comments to elements or interactions of the displayed network directly from the display area.

MINERVA features a built-in interface to DrugBank^25^ and CHEMBL^26^ databases. The ‘Drug’ tab provides an additional query interface, accepting names of drugs as search items, which are used as a query to DrugBank and CHEMBL. The retrieved drug targets are displayed on the visualized network. Users can search for multiple, comma-separated terms, allowing to look for elements subject to potential drug-drug interaction. Figure 1a illustrates a query for two Parkinson’s disease drugs, levodopa and carbidopa, with their targets displayed in the Parkinson’s disease map (see the section ‘MINERVA use cases’). One can see the semantic zoom areas—gray for pathways, colored for compartments—automatically generated by the platform.

#### Export of explored diagrams

The hosted content can be easily exported into different formats. Users can select an area of the visualized content and download the contents either as an image or as a model. The latter functionality is of particular importance, as the downloaded model can be opened directly by a corresponding SBGN editor, and contains all annotations available in the online version. Thus, the users can directly work with the contents published on a given MINERVA instance. Moreover, hosted content can be directly exported, either as a tab-delimited file or as a network. These functionalities ensure high accessibility and re-use of the curated content. Figure 1b illustrates export of a portion of the Parkinson’s disease map into a CellDesigner file.

#### Visualization of custom experimental data

The MINERVA platform allows for an upload of experimental datasets. Registered users have access to an additional menu, allowing them to upload and manage experimental results in a simple format. The uploaded data are used to generate a custom overlay by highlighting elements with an indicated intensity and color and assigning line width and line color to interactions. The uploaded dataset may contain different types of elements, allowing visualization of multiple ‘omics’ data types. Figure 1c shows a visualization of a Parkinson’s disease-relevant transcriptome dataset. The bottom part of the left panel shows the ‘User-provided overlays’ menu, allowing for an online upload of datasets by registered users.

#### Feedback to content moderators

Networks displayed on the MINERVA platform are interactive, and allow direct annotation by the user. Right-click in the display area of the map invokes a form, where users can provide their remarks. The comments can be made visible on the map and pinned to a specific element or interaction, and are managed via the administrator’s panel. The right part of Figure 1c shows an interactive menu with the commenting functionality invoked for an element of the map.

### MINERVA use cases

The MINERVA platform is designed to be a standalone knowledge repository, supporting visualization and exploration of networks represented in a SBGN-compliant format. A number of projects are currently powered by the platform, with most notable examples being Parkinson’s disease (PD) map^4^ and AsthmaMap.^27^

#### Parkinson’s disease map

The PD map (http://pdmap.uni.lu) focuses on pathways involved in neurodegenerative processes of the neuronal system, in particular on the degeneration of dopaminergic neurons of substantia nigra pars compacta. Its manually curated interactions are supported by over a thousand publications, resulting in roughly 5,000 elements linked by over 2,000 interactions. The PD map utilizes all MINERVA functionalities to explore a large repository of molecular mechanisms, and enables users to interpret their experimental data and guide content curation via the commenting system.

## Discussion

Cross-linking of multiple sources of information, and their proper visualization in the context of well-curated knowledge is key for cutting through the complexity of biological systems. MINERVA enables this overlay and exploration, providing both users and curators with a tool for better handling complex knowledge. The platform is under constant development, with a number of interesting directions to further improve its integration into the global ecosystem of bioinformatics tools, content curation and visualization.

### Interfacing knowledge repositories and databases

The MINERVA platform reaches out to other knowledge repositories by handling pure SBGN format in parallel with CellDesigner files. Nevertheless, lot of biological knowledge is present in less-stringent format within a vast amount of currently available literature. For instance, to date, PubMed indexes more than 24 million articles. Knowledge bases established by mining available full-text articles or abstracts for molecular relations are a promising target to explore, cross-link and thus contextualize this rich content.

Structured knowledge on molecular interactions is available in large-scale databases like String^28^ or via integrative curation of MIntAct.^6^ Querying these databases may support curators with an unbiased source of standardized information. Importantly, MINERVA could capitalize on efforts in this direction by bridging to PSICQUIC architecture.^5^ Another valuable source of high-quality molecular interaction networks is the newly established NDEx platform.^29^ NDEx is an online commons, allowing users to upload and share networks for analytical purposes. NDEx complements MINERVA functionalities, as it allows advanced querying and analytical interfaces. The possibility of exporting MINERVA-hosted content to NDEx repository would greatly broaden the knowledge exploration capabilities of MINERVA users. Allowing both import from and export to such network-sharing platform would link independent MINERVA instances and further promote data interoperability.

The abovementioned interaction or network repositories provide content abstracted from a visual representation. Graphical layout is a high-quality information, accompanying molecular interactions in repositories like Reactome,^7^ WikiPathways (http://www.wikipathways.org) and others.^30,31^ Novel interfaces need to be designed and developed, allowing to assemble molecular interaction networks from these heterogeneous sources. A common denominator for a graphical layout is needed for this purpose, and SBGN is a good candidate for this purpose.

An existing link to drug-related databases allows adding an additional information layer on top of a molecular network displayed in MINERVA. Interfaces to other database resources, including experimental datasets, like GEO or ArrayExpress,^32^ would allow for further accretion of information sources, leading to refined hypothesis generation. The mapping between several database-specific or commercial identifiers might be addressed by tools such as BridgeDB.^33^

### Visualization capabilities

At present MINERVA displays the SBGN-encoded layout, which is done manually. The curation of graph layout by humans creates an aesthetically pleasing initial layout, but is a difficult and time-consuming process, especially when updates of the underlying data are frequent and result from querying large knowledge bases. Many layout algorithms address general large graphs,^34^ but to our knowledge no effective algorithm was proposed for large complex hypergraphs that could speed up or improve the quality of human curation.

There are many potential research avenues for future work to investigate this problem. The strong structural aspect of hypergraphs, such as the hyperedges connecting multiple sets of nodes, may allow for an approach similar to Topolayout.^35^ The hierarchical nature of the data may support Hierarchical Edge Bundling^36^ to clarify busy network areas. Including multiple layers of omics data, considering the graph as a multilayer network^37^ and visualizing it accordingly could improve end-user comprehension. Another aspect that impacts layout is node duplication, employed by curators, to improve the aesthetics of a graph^38^ by reducing the number of edge crossings or reducing edge length, or for biological purposes. Supporting this process algorithmically would simplify the task of curators, and facilitate the layout process for an automated algorithm.

### Conclusions

We present the MINERVA platform, a standalone webserver allowing to visualize, annotate and manage molecular interaction networks in SBGN-compliant formats. Our platform is easy to set up, and offers unprecedented integration of SBGN data model with external databases and user interface. Web-based, interactive, automatically annotated content can be enhanced and commented by users, and exported in different formats, including SBGN models, network structure or images.

The MINERVA platform allows hosting of different molecular interaction networks in parallel, giving the users and administrators a flexible tool to set up and use a library of different molecular maps. Future development of the platform foresees linking to a broader range of knowledge bases, improved querying and advanced visualization functionalities. With our work we aim to support the development of the growing number of SBGN-based repositories, especially those that are health-oriented.^4,18,39^

## Materials and methods

### Implementation and availability

MINERVA is a web service using the Java Server Faces 2 technology. The server side, including data parsing, integration, annotation and verification, is implemented in Java 8. The platform uses the Postgres SQL database for data storage and the Hibernate framework as a middle layer between the web server and database. The user-web interface is generated using JSF and PrimeFaces. The displayed content is visualized by Google Maps API, dedicated JavaScript and CSS.

MINERVA is available free of charge under Affero GPL 3.0. Commercial use, where source code is not disclosed, requires a separate license. MINERVA can be downloaded as a virtual machine snapshot or as a Docker container from the project website (http://r3lab.uni.lu/web/minerva-website/). Moreover, a Debian package is provided, allowing setup via the ‘apt-get install’ command.

### Web-based approach

**Figure 2:**
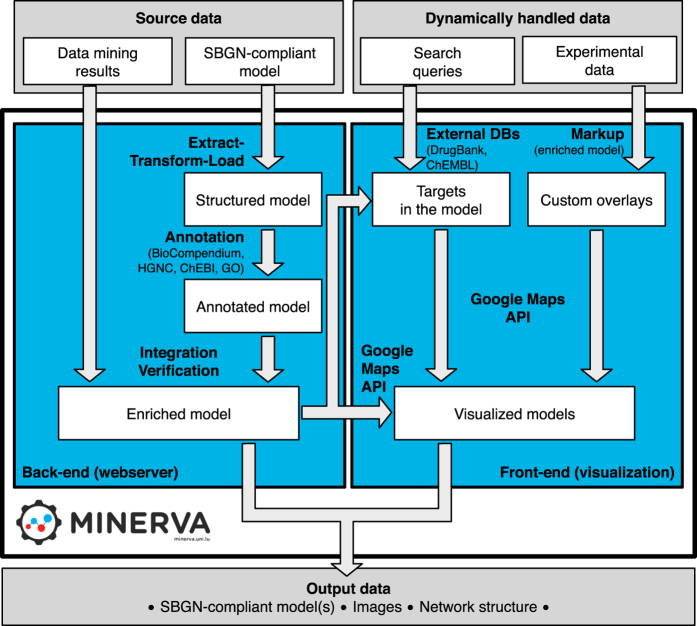
A schematic diagram describing the MINERVA architecture. The back-end—webserver—handles data upload of SBGN-compliant model(s) to the database (‘Structured model’), its annotation (‘Annotated model’) and integration with other provided source files, like pre-defined overlays, overview images and data mining results. The front-end displays the models visualized with the Google Maps API, including overlays based on custom, user-provided datasets. Moreover, the webserver handles search queries, either to the model or to external databases, and visualizes the query results. Finally, a selected part of the model can be exported either as a SGBN-compliant model (.xml file) or as an image (.png or .pdf). A separate view allows exporting network contents or structure.

MINERVA is a web-based platform hosting molecular interaction networks encoded as process diagrams in SBGN and CellDesigner formats. The platform allows direct exploration of the content, including lookup of multiple terms, hyperlinks to available annotations and visualization of high-throughput datasets. Explanatory graphics, like illustration of organs, organelles or pathways, can be displayed and linked with the molecular content of the map, further facilitating the exploration. Each instance of MINERVA platform is an autonomous, standalone webserver. A web-based administration interface allows to manage the hosted content and registered users, independently from the MINERVA development team. Figure 2 summarizes the architecture of MINERVA.

### Content curation in SBGN format

The platform accepts content in SBGN-compliant formats, curated using either CellDesigner software (http://www.celldesigner.org) or SBGN-specific editors (http://sbgn-ed.org). Upon upload, the input file is translated into the data model supported by MINERVA platform. In this regard, MINERVA provides flexibility in the choice of curation tool, and a possibility to translate between formats, as the direct export of the content allows choice between CellDesigner and pure SBGN format (see the section ‘Functionalities for content exploration’).

MINERVA handles, at the moment of publication, 43 different MIRIAM identifiers^40^ (see Supplementary Table 1 for a complete list). Elements or interactions manually annotated with one of these identifiers will be visualized with a hyperlink cross-referencing to the MIRIAM-indicated database. Importantly, a number of MIRIAM identifiers, when provided in the CellDesigner file, trigger automated annotation of the annotated element.
